# The prognostic value of platelet-to-lymphocyte ratio on in-hospital mortality in admitted adult traffic accident patients

**DOI:** 10.1371/journal.pone.0233838

**Published:** 2020-06-17

**Authors:** Sion Jo, Taeoh Jeong, Jae Baek Lee, Youngho Jin, Jaechol Yoon, Boyoung Park

**Affiliations:** 1 Department of Emergency Medicine, College of Medicine, Chonbuk National University, Jeonju-si, Republic of Korea; 2 Research Institute of Clinical Medicine of Chonbuk National University, Jeonju-si, Republic of Korea; 3 Biomedical Research Institute of Chonbuk National University Hospital, Jeonju-si, Republic of Korea; 4 Departmetn of Emergency Medicine, Veterans Health Service Medical Center, Seoul, Republic of Korea; 5 Department of Medicine, College of Medicine, Hanyang University, Seoul, Republic of Korea; National Yang-Ming University, TAIWAN

## Abstract

**Background:**

The predictive value of platelet-to-lymphocyte ratio (PLR) in acute illness is well known, but further evaluation is needed in traffic accident patients.

**Methods:**

This retrospective observational study enrolled consecutive adult patients involved in traffic accidents who were admitted to the study hospital’s emergency department during 1 year. The initial platelet and lymphocyte counts after arrival at the emergency department were the variables of interest. The primary outcome was in-hospital mortality. Data on baseline characteristics, comorbidities, and physiological and laboratory variables were collected. Multivariate Cox proportional hazard modelings were used to identify the variables independently associated with the outcome.

**Results:**

A total of 1,522 traffic accident patient were screened, and 488 patients were enrolled. In all, 43 (8.8%) patients died in the hospital. The median PLR was 115.3 (interquartile range 71.3;181.8). The in-hospital mortality rate of the 1^st^ tertile of PLR (21.5%) was significantly higher than the rates of the 2^nd^ (2.5%) and 3^rd^ (2.5%) tertiles. The area under the receiver operating characteristic curve of PLR for in-hospital survival was 0.82 (95% confidential interval [CI], 0.74–0.89), which was greater than that of lymphocyte count (0.72; 95% CI 0.63–0.81) and platelet count (0.67; 95% CI 0.57–0.76). The Kaplan-Meier curves showed a significant difference in survival between the tertiles (p<0.001). The Cox regression model showed that the 2^nd^ tertile of PLR was independently associated with lower in-hospital mortality (adjusted hazard ratio 0.30; 95% CI, 0.09–0.98), compared to the 1^st^ tertile.

**Conclusion:**

PLR was significantly associated with an increased risk of in-hospital mortality in admitted adult traffic accident patients.

## Introduction

### Background

Trauma is one of the major health problems worldwide. [1] In 2013, 973 million people were estimated to have experienced trauma, 56.2 million patients were admitted to the hospital due to trauma, and 8 million people died from trauma globally. [2] Therefore, prognostication in trauma care is of interest to physicians. While traditional risk scores, such as the Injury Severity Score (ISS), [3] Revised Trauma Score (RTS), [4] and TRauma and Injury Severity Score (TRISS) [5] are widely used, researchers have been keen to identify more prognostic factors in trauma patients.

Platelet-to-lymphocyte ratio (PLR) has emerged as a prognostic marker in various acute conditions, including sepsis, [6] myocardial infarction, [7] pulmonary embolism, [8] acute heart failure, [9] acute exacerbation of chronic obstructive pulmonary disease, [10,11] gastrointestinal bleeding, [12] pancreatitis, [13] and diabetic ketoacidosis. [14] However, the literature on its use in trauma care is insufficient. PLR has been evaluated in low-energy trauma patients [15] and a small number of critically ill patients with severe trauma. [16] Therefore, the present study aimed to evaluate the prognostic value of PLR in predicting in-hospital mortality in patients involved in traffic accidents, which were considered as one of the main components of major trauma. [17,18] We hypothesized that the initial PLR after arrival at the emergency department (ED) was associated with an increased risk of in-hospital mortality in admitted adult traffic accident patients.

## Materials and methods

### Study design and setting

The present study was a retrospective chart review study and was approved by the institutional review board (IRB) of Chonbuk National University Hospital. The IRB waived the requirement for informed consent in the present study. The study hospital is a 1200-bed urban, academic, tertiary care, university hospital. The study hospital’s ED is the largest referral center in the province. We referred to the Standards for the Reporting of Diagnostic Accuracy recommendations when analyzing the results. [19,20] In the study hospital’s ED, initial blood tests were usually performed within few minutes after presentation to the ED.

### Selection of participants

The study hospital’s ED is part of the Emergency Department-based Injury In-depth Surveillance (EDIIS). The EDIIS is a nationwide injury database that was developed and operated by the Korea Centers for Disease Control and Prevention. Among the patients who visited the study hospital’s ED between January 1, 2016, and December 31, 2016, patients who were registered as involved in traffic accidents were screened. Among those, patients who were admitted to the study hospital were eligible for inclusion in the study. However, we excluded the following patients: (1) pediatric patients aged below 18 years and (2) patients who had no ED laboratory test results.

### Data collection

The EDIIS database reports data on a total of 246 variables, which includes basic demographics, injury epidemiologic information, comorbidities, initially assessed clinical findings at ED, diagnosis, disposition at the ED, and patient outcome after admission. For the present study, data of the following variables were extracted: age, sex, emergency medical service (EMS) use, transfer from other hospitals or facilities, comorbidities (hypertension, diabetes mellitus, coronary artery disease, cerebrovascular disease, or malignancy), time to ED arrival, physiology at ED presentation (systolic blood pressure [SBP], diastolic blood pressure, heart rate, respiratory rate [RR], body temperature [BT], Glasgow Coma Scale [GCS] score), patient status (car vs. motorcycle vs. bicycle vs. pedestrian), counter status (car vs. motorcycle or bicycle status vs. object vs. unknown), injury site (brain, head/face/neck, chest, abdomen, extremity, spine), ISS, intensive care unit (ICU) admission, emergent operation, total operation, time to admission, time to hospital discharge after admission, and survival status at hospital discharge. Data on platelet and lymphocyte counts were also extracted. The initial platelet count and lymphocyte count after ED arrival were selected. RTS, TRISS, and PLR were calculated using the collected data.

### Outcome measures

The primary outcome was in-hospital mortality.

### Statistical analysis

The data were analyzed for normal distribution of continuous variables using the Shapiro-Wilk test. Continuous data were presented as medians and interquartile ranges (IQRs) if they were distributed non-normally and as mean and standard deviation if they were distributed normally. Categorical data were presented as count and percentage.

Comparison of normally distributed data was performed using an independent sample t-test. For non-normally distributed data, comparisons were performed using the Mann-Whitney U test or Kruskal-Wallis test. For categorical data, the chi-squared test was performed. If necessary, a chi-squared test with a Fischer’s exact test for 2×2 table was performed. Results were considered significant at a threshold of p<0.05 (two-tailed). Differences were tested between the survivor group and non-survivor group and between PLR tertiles.

The predictive capacities of the PLR, platelet count, and lymphocyte count with regard to the primary outcome were assessed by area under the receiver operating characteristic (AUROC) curve analysis. The standard errors of the mean and p-values for the AUROC curves were calculated and compared using the methods proposed by Hanley and McNeil [21]. [19] Sensitivity (SN), specificity (SP), positive likelihood ratio (+LR), and negative likelihood ratio (-LR) were evaluated at a clinical cutoff value.

The variables that were found to be statistically associated with the primary outcome based on univariate analysis using Cox regression were entered into the multivariate regression models. Multivariate Cox hazard regression analysis was performed to determine the independent effect of PLR on the primary outcome. The results of the Cox regression analysis were reported as hazard ratios (HRs) and 95% confidence intervals (CIs). The Kaplan-Meier survival analysis was performed to show the cumulative survival for each PLR tertile.

To reduce imbalance and achieve comparable distribution of severity between survivors and non-survivors for an unbiased estimation of PLR on survival, we applied 1:1 statistical matching of survivors and non-survivors in a nested case-control setting. The ISS, RTS, and TRISS which are widely applied to measure the injury severity of trauma patients were included as matching variables. We considered both the time to death and censoring in the total data analysis, thus Cox hazard regression analysis was applied. While, logistic regression analysis was applied for this nested case-control study to propose significantly associated variables as general analysis for case-control study and the association between PLR and survival was confirmed again.

All analyses were performed using an R software package (version 3.4.3, 2015; R Core Team, Vienna, Austria), Stata 11.1 (StataCorp LP, TX, USA), and Statistical Analysis System (SAS) 9.1 (SAS Institute Inc., Cary, NC, USA).

## Results

**Fig 1:**
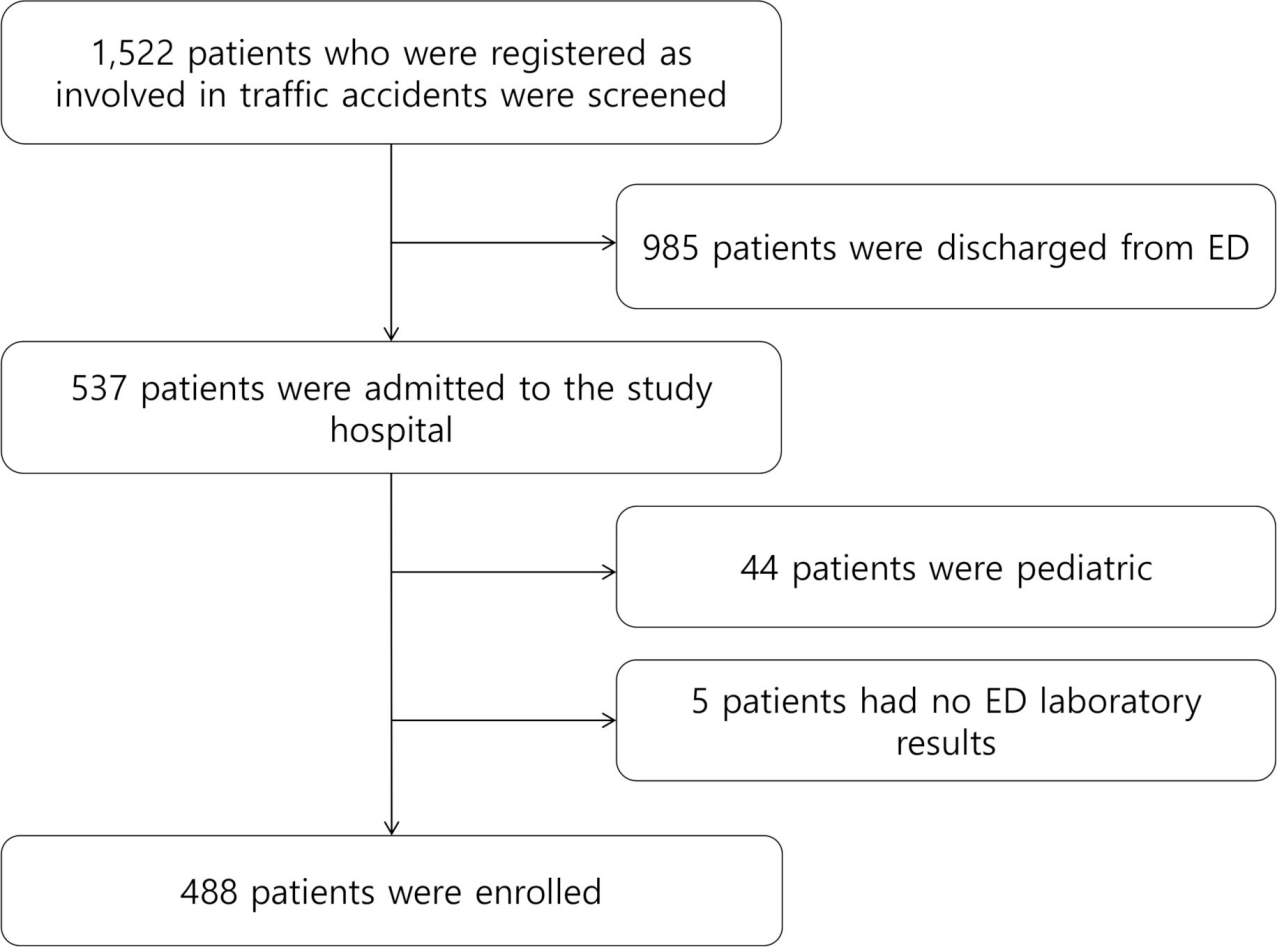
The Standards for the Reporting of Diagnostic Accuracy flow diagram for the study.

In this study, 1,522 traffic accident patients were screened. Among them, 537 patients were admitted to the study hospital. A total of 44 pediatric patients and five patients with no ED laboratory test results were excluded. Finally, 488 patients were enrolled in the analysis (Fig 1).

Table 1 shows the baseline characteristics of the enrolled patients before and after matching. Before matching, most patients were involved in car accidents (n = 234, 48.0%), followed by motorcycle accidents (n = 103, 21.1%), and pedestrian accidents (n = 99, 20.3%). Cars were most frequently encountered in the accidents (n = 263, 53.9%), followed by inanimate objects (n = 177, 36.3%). The extremity was the most frequent anatomical site (n = 226, 46.3%), followed by the spine (n = 172, 35.3%), head/face/neck (n = 149, 30.5%), brain (n = 143, 29.3%), chest (n = 138, 28.3%), and abdomen (n = 68, 13.9%). A total of 147 patients (30.1%) were admitted to the ICU, and 143 patients (29.3%) underwent emergent operation. The median platelet and lymphocyte counts were 222.5 (183.0, 265.0) and 1.8 (1.2, 3.0), respectively. The median PLR was 115.3 (71.3, 181.8).

**Table 1:** Baseline characteristics of the enrolled patients, comparison between survivor and non-survivor groups, and 1:1 propensity score matching (PSM) of patients.

Variable	Total	Total no. of patients		p-value	Patients after PSM		p-value
		Survivor group	Non-survivor group		Survivor group	Non-survivor group	
Number	488 (100%)	445 (91.2%)	43 (8.8%)		43 (50.0%)	43 (50.0%)	
Age	60 [45.5;72.0]	59.0 [45.0;71.0]	66.0 [55.5;75.0]	0.019	64.0 [53.5;73.0]	66.0 [55.5;75.0]	0.492
Male sex	333 (68.2%)	303 (68.1%)	30 (69.8%)	0.957	27 (62.8%)	30 (69.8%)	0.648
EMS use	266 (54.5%)	233 (52.4%)	33 (76.7%)	0.004	29 (67.4%)	33 (76.7%)	0.471
Transfer	62 (12.7%)	60 (13.5%)	2 (4.7%)	0.155	4 (9.3%)	2 (4.7%)	0.672
HTN	134 (27.5%)	124 (27.9%)	10 (23.3%)	0.640	14 (32.6%)	10 (23.3%)	0.471
DM	61 (12.5%)	54 (12.1%)	7 (16.3%)	0.587	5 (11.6%)	7 (16.3%)	0.756
CAD	30 (6.2%)	27 (6.1%)	3 (7.0%)	1.000	2 (4.7%)	3 (7.0%)	1.000
CVA	24 (4.9%)	23 (5.2%)	1 (2.3%)	0.650	6 (14.0%)	1 (2.3%)	0.115
Malignancy	23 (4.7%)	21 (4.7%)	2 (4.7%)	1.000	1 (2.3%)	2 (4.7%)	1.000
Time to ED, min	83 [40.5;208.0]	92 [44;215]	38 [26.5;101.5]	<0.001	54.0 [37.0;122.5]	38.0 [26.5;101.5]	0.111
SBP	130.0 [110.0;150.0]	130.0 [113.0;150.0]	95.0 [70.0;125.0]	<0.001	110.0 [78.5;130.0]	95.0 [70.0;125.0]	0.132
DBP	78.0 [50.6;90.0]	80.0 [67.0;90.0]	60.0 [45.0;80.0]	<0.001	70.0 [50.0;80.0]	60.0 [45.0;80.0]	0.123
PR	84.0 [74.0;95.5]	84.0 [74.0;94.0]	90.0 [75.0;113.5]	0.045	84.0 [73.5;106.0]	90.0 [75.0;113.5]	0.551
RR	18.0 [18.0;20.0]	18.0 [18.0;20.0]	18.0 [18.0;20.5]	0.871	18.0 [18.0;21.0]	18.0 [18.0;20.5]	0.721
BT	36.5 [36.1;36.9]	36.5 [36.2;36.9]	36.0 [35.5;36.5]	<0.001	36.0 [36.0;36.5]	36.0 [35.5;36.5]	0.238
GCS	15.0 [15.0;15.0]	15.0 [15.0;15.0]	7.0 [3.0;13.5]	<0.001	11.0 [6.0;15.0]	7.0 [3.0;13.5]	0.045
Patient status				0.054			0.889
Car	234 (48.0%)	222 (49.9%)	12 (27.9%)		14 (32.6%)	12 (27.9%)	
Motorcycle	103 (21.1%)	90 (20.2%)	13 (30.2%)		14 (32.6%)	13 (30.2%)	
Bicycle	52 (10.7%)	46 (10.3%)	6 (14.0%)		4 (9.3%)	6 (14.0%)	
Pedestrian	99 (20.3%)	87 (19.6%)	12 (27.9%)		11 (25.6%)	12 (27.9%)	
Counter status				0.077			0.078
Car	263 (53.9%)	232 (52.1%)	31 (72.1%)		22 (51.2%)	31 (72.1%)	
Motor or bicycle	7 (1.4%)	7 (1.6%)	0 (0.0%)		0 (0.0%)	0 (0.0%)	
Object	177 (36.3%)	165 (37.1%)	12 (27.9%)		19 (44.2%)	12 (27.9%)	
Unknown	41 (8.4%)	41 (9.2%)	0 (0.0%)		2 (4.7%)	0 (0.0%)	
Injury site							
Brain	143 (29.3%)	115 (25.8%)	28 (65.1%)	<0.001	33 (76.7%)	28 (65.1%)	0.342
Head/Face/Neck	149 (30.5%)	144 (32.4%)	5 (11.6%)	0.008	20 (46.5%)	5 (11.6%)	0.001
Chest	138 (28.3%)	119 (26.7%)	19 (44.2%)	0.025	14 (32.6%)	19 (44.2%)	0.375
Abdomen	68 (13.9%)	50 (11.2%)	18 (41.9%)	<0.001	10 (23.3%)	18 (41.9%)	0.107
Extremity	226 (46.3%)	215 (48.3%)	11 (25.6%)	0.007	16 (37.2%)	11 (25.6%)	0.353
Spine	172 (35.3%)	156 (35.1%)	16 (37.2%)	0.908	18 (41.9%)	16 (37.2%)	0.825
ISS	27.0 [16.0;48.0]	26.0 [16.0;41.0]	66.0 [44.5;70.5]	<0.001	66.0 [50.0;75.0]	66.0 [44.5;70.5]	0.428
RTS	11.0 [11.0;11.0]	11.0 [11.0;11.0]	8.2 [7.2;10.0]	<0.001	9.5 [8.4;10.6]	8.2 [7.2;10.0]	0.025
TRISS	99.0 [95.2;99.8]	99.3 [97.1;99.8]	60.0 [17.7;83.8]	<0.001	72.8 [54.4;82.6]	60.0 [17.7;83.8]	0.107
ICU admission	147 (30.1%)	106 (23.8%)	41 (95.3%)	<0.001	31 (72.1%)	41 (95.3%)	0.009
Emergent op	143 (29.3%)	113 (25.4%)	30 (69.8%)	<0.001	17 (39.5%)	30 (69.8%)	0.009
Total op	311 (63.7%)	279 (62.7%)	32 (74.4%)	0.127	29 (67.4%)	32 (74.4%)	0.635
ED LOS, h	9.0 [4.0;17.0]	9.0 [4.0;17.0]	10.0 [4.0;21.0]	0.643	8.0 [3.0;16.0]	10.0 [4.0;21.0]	0.375
Hospital LOS, days	13.0 [7.0;26.0]	14.0 [8.0;27.0]	2.0 [1.0;8.0]	<0.001	38.0 [16.0;68.5]	2.0 [1.0;8.0]	<0.001
Platelets	222.5 [183.0;265.0]	227.0 [188.0;265.0]	183.0 [141.0;230.0]	<0.001	198.0 [157.0;265.0]	183.0 [141.0;230.0]	0.021
Lymphocytes	1.8 [1.2;3.0]	1.8 [1.2;2.8]	3.4 [2.0;5.4]	<0.001	2.6 [1.5;4.8]	3.4 [2.0;5.4]	0.130
PLR	115.3 [71.3;181.8]	124.2 [79.5;187.2]	51.3 [32.3;77.9]	<0.001	85.8 [57.6;137.2]	51.3 [32.3;77.9]	<0.001

Abbreviations PSM, propensity score matching; EMS, emergency medical service; HTN, hypertension; DM, diabetes mellitus; CAD, coronary artery disease; CVA, cerebrovascular disease; ED, emergency department; SBP, systolic blood pressure; DBP, diastolic blood pressure; PR, pulse rate; RR, respiratory rate; BT, body temperature; GCS, Glasgow coma scale; ISS, injury severity scale; RTS, revised trauma score; TRISS, trauma and injury severity score; ICU, intensive care unit; op, operation; LOS, length of stay; PLR, platelet to lymphocyte ratio.

Those in the non-survivor group were older, used EMS more often, and arrived earlier from the scene to the ED than those in the survivor group. The initial ED physiological variables except RR were poor in the non-survivor group than that of the survivor group. In-hospital mortality rates were the highest for motorcycle accidents, followed by car and pedestrian accidents. The non-survivor group showed a higher prevalence of brain, chest, and abdominal injuries and lower prevalence of head/face/neck and extremity injuries than the survivor group. The risk scores, including ISS, RTS, and TRISS, worse in the non-survivor group than in the survivor group. The platelet count was lower and lymphocyte count was higher in the non-survivor group than in the survivor group. The PLR was lower in the non-survivor group than in the survivor group (51.3 [32.3, 77.9] vs. 124.2 [79.5, 187.2], p<0.001).

**Fig 2:**
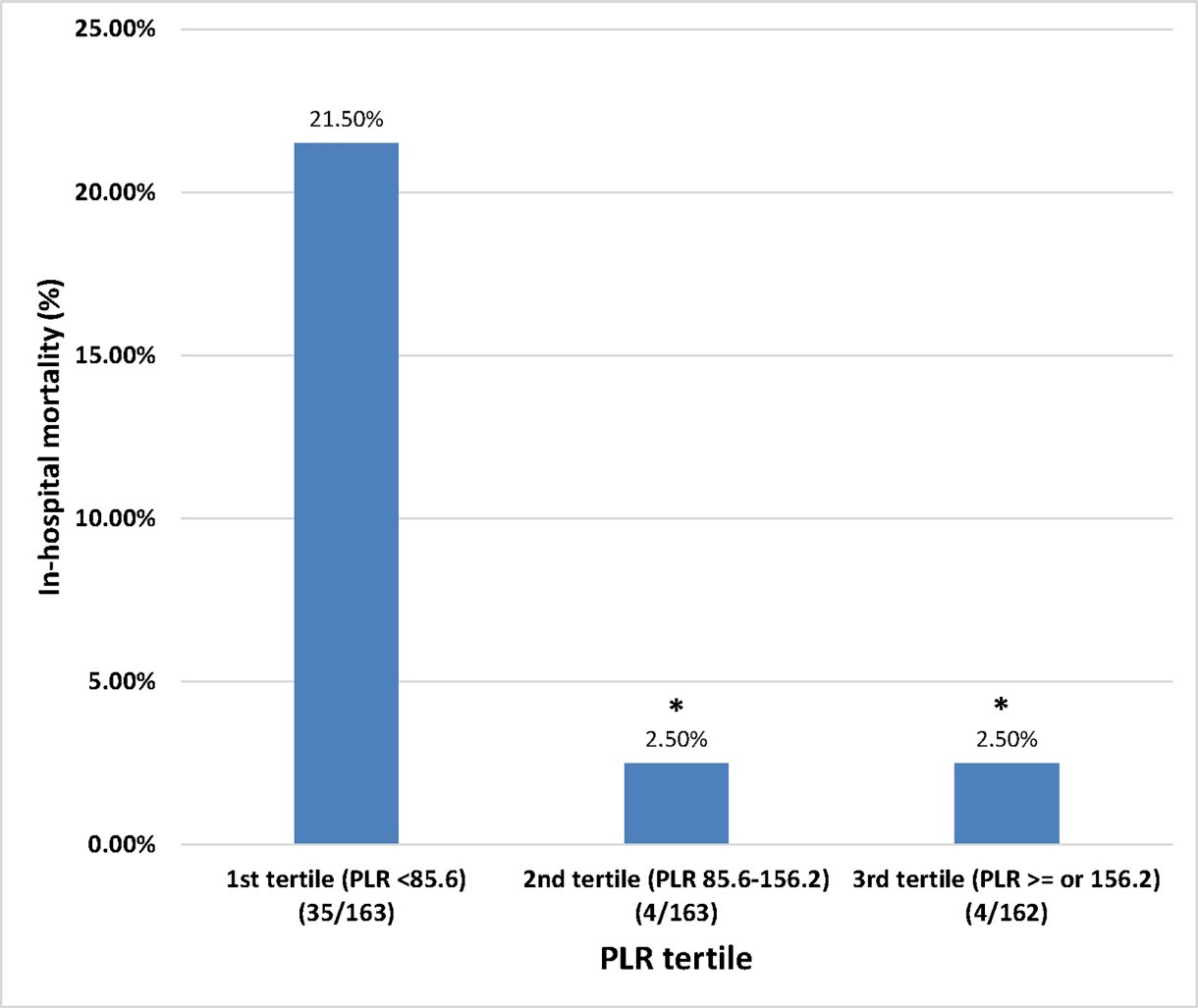
In-hospital mortality according to tertile of platelet-to-lymphocyte ratio (PLR).

PLR ranged from 21.5 to 972.2. For further analysis, PLR was divided into tertile. The cutoff values were 85.6 and 156.2. The in-hospital mortality rates of the 1^st^, 2^nd^, and 3^rd^ tertile were 21.5% (35/163), 2.5% (4/163), and 2.5% (4/162), respectively. The in-hospital mortality rate of the 1st tertile was significantly higher than the rates of the 2^nd^ and 3^rd^ tertiles (both p < 0.001) (Fig 2). When the PLR was below 85.6, the SN, SP, +LR, and -LR were 90.7%, 35.5%, 1.41, and 0.26, respectively.

After matching for ISS, RTS, and TRISS, most of the characteristics between the survivors and non-survivors were comparable, except GCS score, injury site for head/face/neck, RTS, ICU admission, emergent operation, hospital LOS, platelet count, and PLR which was our target variable (Table 1).

The AUROC value of PLR for in-hospital survival was 0.82 (95% CI, 0.74–0.89). The AUROC values of platelet count and lymphocyte count for in-hospital mortality were 0.67 (0.57–0.76) and 0.72 (0.63–0.81), respectively. The AUROC value of PLR was significantly higher than those of platelet count (p = 0.001) and lymphocyte count (p = 0.033). There was no significant difference between the AUROC values of platelet and lymphocyte counts (p = 0.251).

**Fig 3:**
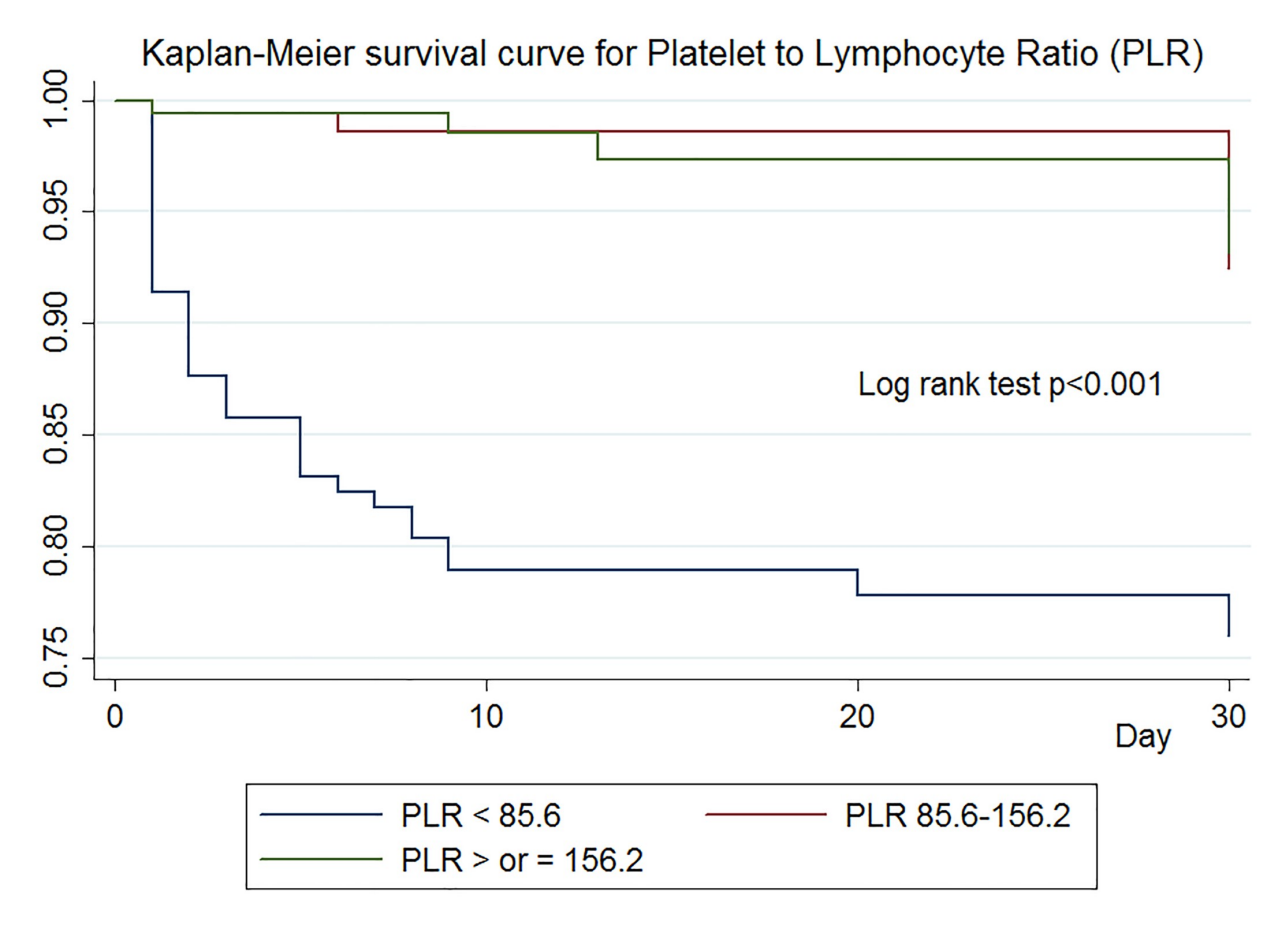
Kaplan-Meier survival curves according to tertiles of platelet-to-lymphocyte ratio (PLR).

The multivariate Cox regression models (Table 2), adjusted for age, EMS use, SBP, RR, BT, GCS score, patient status, injury site, ISS, RTS, TRISS, ICU admission, and emergent operation, revealed that the 2^nd^ tertile of PLR was independently predictive of in-hospital mortality (AHR, 0.30 [0.09–0.98]; p = 0.047). Age, abdominal injury, ICU admission, and emergent operation remained significant variables. The Kaplan-Meier survival analysis showed a significant difference between tertiles (log-rank test p < 0.001) (Fig 3). After propensity score matching, the 2^nd^ tertile of PLR was found to remain an independently associated factor of in-hospital mortality with a similar HR as that in the total population (AOR, 0.21 [0.05–0.97]; p = 0.046). Other variables, except injury site and emergent operation which showed a significant association with in-hospital mortality in the total population were not significant in the matched population (Table 3).

**Table 2:** Cox hazard regression analysis for the total number of patient.

Variable	Unadjusted hazard ratio	p-value	Adjusted hazard ratio	p-value
Age	1.02 (1.00–1.04)	0.058	1.05 (1.01–1.08)	0.014
Male sex	1.05 (0.55–2.01)	0.883		
EMS use	2.63 (1.29–5.36)	0.008	2.05 (0.85–4.96)	0.112
Transferred	0.34 (0.08–1.40)	0.134		
HTN	0.79 (0.39–1.60)	0.507		
DM	1.27 (0.57–2.86)	0.561		
CAD	1.24 (0.38–4.00)	0.724		
CVA	0.43 (0.06–3.11)	0.401		
Malignancy	1.08 (0.26–4.49)	0.912		
Time to ED, min	1.00 (1.00–1.00)	0.301		
SBP	0.97 (0.97–0.98)	<0.001	1.00 (0.98–1.01)	0.546
DBP*	0.96 (0.95–0.97)	<0.001	Omitted	
PR	1.01 (0.99–1.02)	0.303		
RR	0.88 (0.82–0.93)	<0.001	1.07 (0.92–1.23)	0.378
BT	0.91 (0.88–0.94)	<0.001	1.00 (0.94–1.07)	0.929
GCS	0.75 (0.70–0.80)	<0.001	0.94 (0.79–1.13)	0.519
Patient				
Car	Reference		Reference	
Motorcycle	2.43 (1.11–5.32)	0.027	1.08 (0.41–2.84)	0.884
Bicycle	2.39 (0.89–6.39)	0.082	1.01 (0.28–3.70)	0.986
Pedestrian	2.12 (0.95–4.76)	0.068	1.46 (0.57–3.79)	0.432
Counter				
Car	Reference			
Motor or bicycle	Omitted			
Object	0.60 (0.31–1.18)	0.138		
Unknown	Omitted			
Injury site				
Brain	4.17 (2.22–7.84)	<0.001	1.81 (0.63–5.22)	0.271
Head/Face/Neck	0.29 (0.12–0.75)	0.010	0.47 (0.16–1.41)	0.179
Chest	1.97 (1.07–3.60)	0.028	1.20 (0.49–2.93)	0.690
Abdomen	4.70 (2.54–8.68)	<0.001	3.80 (1.50–9.64)	0.005
Extremity	0.35 (0.18–0.70)	0.003	0.78 (0.30–2.05)	0.617
Spine	1.08 (0.58–2.01)	0.807		
ISS	1.06 (1.04–1.07)	<0.001	1.01 (0.97–1.05)	0.589
RTS	0.68 (0.63–0.73)	<0.001	0.71 (0.41–1.26)	0.250
TRISS	0.96 (0.95–0.97)	<0.001	1.01 (0.98–1.04)	0.615
ICU admission	46.2 (11.1–191.2)	<0.001	6.66 (1.30–34.13)	0.023
Emergent op	5.26 (2.73–10.12)	<0.001	2.84 (1.24–6.54)	0.014
Total op	1.34 (0.67–2.68)	0.406		
ED LOS, h	1.00 (0.98–1.03)	0.774		
Platelets	0.99 (0.99–0.99)	<0.001	Omitted	
Lymphocytes	1.52 (1.32–1.75)	<0.001	Omitted	
PLR	0.98 (0.97–0.99)	<0.001	Omitted	
PLR 1^st^ tertile	Reference		Reference	
PLR 2^nd^ tertile	0.12 (0.04–0.33)	<0.001	0.30 (0.09–0.98)	0.047
PLR 3^rd^ tertile	0.12 (0.04–0.33)	<0.001	0.71 (0.20–2.53)	0.596

Abbreviations EMS, emergency medical service; HTN, hypertension; DM, diabetes mellitus; CAD, coronary artery disease; CVA, cerebrovascular disease; ED, emergency department; SBP, systolic blood pressure; DBP, diastolic blood pressure; PR, pulse rate; RR, respiratory rate; BT, body temperature; GCS, Glasgow coma scale; ISS, injury severity scale; RTS, revised trauma score; TRISS, trauma and injury severity score; ICU, intensive care unit; op, operation; LOS, length of stay; PLR, platelet to lymphocyte ratio.

* DBP showed high collinearity with SBP.

**Table 3:** Logistic regression analysis of the 1:1 propensity score matched patients.

Variable	Unadjusted odd ratio	p-value	Adjusted odd ratio	p-value
Age	1.01 (0.98–1.03)	0.547		
Male sex	1.37 (0.56–3.36)	0.494		
EMS use	1.59 (0.61–4.13)	0.338		
Transferred	0.48 (0.08–2.75)	0.406		
HTN	0.63 (0.24–1.63)	0.338		
DM	1.48 (0.43–5.08)	0.535		
CAD	1.54 (0.24–9.69)	0.647		
CVA	0.15 (0.02–1.28)	0.082	0.13 (0.01–1.60)	0.111
Malignancy	1.00 (0.13–7.44)	1.000		
Time to ED, min	1.00 (1.00–1.00)	0.603		
SBP	0.99 (0.98–1.00)	0.081	0.99 (0.98–1.01)	0.520
DBP*	0.98 (0.97–1.00)	0.073	Omitted	
PR	1.00 (0.99–1.01)	0.801		
RR	0.93 (0.83–1.04)	0.183		
BT	0.83 (0.48–1.43)	0.504		
GCS	0.92 (0.84–1.01)	0.066	1.04 (0.83–1.31)	0.720
Patient status				
Car	Reference			
Motorcycle	1.38 (0.30–6.20)	0.679		
Bicycle	0.85 (0.28–2.59)	0.777		
Pedestrian	0.79 (0.26–2.42)	0.674		
Counter status				
Car	Reference			
Motor or bicycle	Omitted			
Object	0.45 (0.18–1.11)	0.083	0.31 (0.08–1.20)	0.091
Unknown	Omitted			
Injury site				
Brain	0.57 (0.22–1.46)	0.238		
Head/Face/Neck	0.15 (0.05–0.46)	0.001	0.11 (0.02–0.55)	0.007
Chest	1.64 (0.68–3.94)	0.269		
Abdomen	2.38 (0.94–6.03)	0.069	1.65 (0.43–6.34)	0.467
Extremity	0.58 (0.23–1.46)	0.247		
Spine	0.82 (0.35–1.96)	0.659		
ISS	0.99 (0.96–1.02)	0.409		
RTS	0.75 (0.59–0.95)	0.019	0.93 (0.52–1.65)	0.796
TRISS	0.98 (0.97–1.00)	0.038	1.00 (0.97–1.02)	0.687
ICU admission	7.94 (1.65–38.06)	0.010	3.57 (0.43–29.91)	0.241
Emergent op	3.53 (1.45–8.62)	0.006	3.81 (1.07–13.55)	0.039
Total op	1.40 (0.55–3.58)	0.477		
ED LOS, h	1.03 (0.98–1.09)	0.197		
Platelets	0.99 (0.99–1.00)	0.017	Omitted	
Lymphocytes	1.20 (0.97–1.49)	0.101	Omitted	
PLR	0.99 (0.98–1.00)	0.016	Omitted	
PLR 1^st^ tertile	Reference		Reference	
PLR 2^nd^ tertile	0.16 (0.05–0.55)	0.003	0.21 (0.05–0.97)	0.046
PLR 3^rd^ tertile	0.34 (0.09–1.31)	0.118	2.33 (0.31–17.78)	0.415

Abbreviations EMS, emergency medical service; HTN, hypertension; DM, diabetes mellitus; CAD, coronary artery disease; CVA, cerebrovascular disease; ED, emergency department; SBP, systolic blood pressure; DBP, diastolic blood pressure; PR, pulse rate; RR, respiratory rate; BT, body temperature; GCS, Glasgow coma scale; ISS, injury severity scale; RTS, revised trauma score; TRISS, trauma and injury severity score; ICU, intensive care unit; op, operation; LOS, length of stay; PLR, platelet to lymphocyte ratio.

* DBP showed high collinearity with SBP.

## Discussion

The present study found that PLR is an independent predictive marker in adult traffic accident patients. Even after propensity score matching, PLR showed significance, despite the small sample size. To the best of our knowledge, this is the first study that evaluates the predictive value of PLR in adult traffic accident patients, which is considered as one of the main components of major trauma.

Trauma results in significant morbidity and mortality, which warrants the risk prediction for the trauma patients. The well-used risk scores such as the ISS, RTS, and TRISS are based on physiology and injury site. Moreover, several laboratory markers have been tested for their prognostic value for mortality such as hemoglobin, creatinine, pH, base excess, activated partial thromboplastin time, prothrombin time/international normalized ratio, and lactate. [22, 23] Recently, new risk scoring systems that incorporate physiology and laboratory markers have been introduced. [22, 24]

There are notable advantages of using PLR. PLR is very simple and easy to calculate. Furthermore, PLR can be used in almost all EDs worldwide, including those in developing countries, because the complete blood count (CBC) test is widely used and is very cheap. Moreover, CBC can be determined rapidly. The time taken per analysis is a few minutes when using recent commercial automated hematology analyzers. Thus, PLR can be available in the early phase while treating acutely ill patients. Thus, several studies have been conducted to determine the predictive performance of PLR in various acute illnesses.

We found significant gaps in mortality rates between PLR tertiles (21.5% at the 1^st^ tertile vs. 2.5% each at the 2^nd^ and 3^rd^ tertiles). The Kaplan-Meier curve showed that the gap in mortality rates was predominant in the early phase (in 10 days). Furthermore, PLR was significantly associated with in-hospital mortality after adjusting for the baseline characteristics, comorbidities, physiology, injury site, and traditional risk scores. Even the ISS, RTS, and TRISS became insignificant in the multivariate Cox regression model.

Platelets play a key role in hemostasis; they rapidly bind to damaged blood vessels and aggregate to form thrombi, thus preventing excessive bleeding. [25, 26] Moreover, platelets play an important role in the development of sepsis. [27] Platelet activation leads to endothelial injury and promotes neutrophil extracellular trap and microthrombi formation. This exacerbates septic coagulation and inflammatory reactions. Furthermore, disseminated intravascular coagulation leads to multi-organ dysfunction syndrome (MODS). Some studies have suggested a direct link between low platelet count and MODS in trauma patients. [28, 29] Additionally, some studies have suggested that platelets induce the release of inflammatory cytokines [30] and interact with neutrophils, T cells, and macrophages. [31]

Lymphocytes are the major cellular components of the humoral and cell-mediated immune system and include T cell, B cells, and natural killer cells. [32] The immune competence of lymphocytes is negatively modulated after trauma or hemorrhage, and a decrease in lymphocyte counts is associated with the development of sepsis and MODS among trauma patients. [33–35] The normal range of PLR has not been confirmed yet, but the mean PLR in healthy adults in South Korea is reported to be 132.40 ± 43.68. [36] The reference ranges for platelet and lymphocyte counts are 150,000 to 450,000 and 1.0 to 3.0, respectively. In the present study, the 1st tertile of PLR (<85.6) showed significantly higher mortality than the other two tertiles. The median values of platelet and lymphocyte counts in enrolled patients, the survivor group, and the non-survivor group were within the reference ranges, except in the case of lymphocyte counts in the non-survivor group. Platelet counts were significantly lower in the non-survivor group than those in the survivor group; on the contrary, lymphocyte counts were significantly higher in the non-survivor group than those in the survivor group. The Kaplan-Meier curve showed that a survival gap between the groups was predominant within several days after trauma. Thus, we hypothesized that the notable prognostic value of PLR, which is significantly higher than both platelet and lymphocyte counts, was a comprehensive reflection of the extent to which the trauma affected the two variables (platelet and lymphocyte counts).

Recently, few studies have evaluated the PLR performance in trauma patients. Emektar et al. investigated the effect of PLR at hospital admission on 1-year mortality in 560 elderly patients with hip fractures. [15] The platelet count was similar between the non-survivor and survivor groups (214 [IQR, 172–257] vs. 206 [159–263], p = 0.3). The lymphocyte count was lower in the non-survivor group than in the survivor group (1.0 [IQR, 0.7–1.4] vs. 1.2 [0.9–1.6], p = 0.004). PLR was higher in the non-survivor group than in the survivor group (197 [IQR, 140–289] vs. 178 [119–248], p = 0.02). These findings are contrary to our findings. In our study, the platelet count was lower, lymphocyte count was higher, and PLR was lower in the non-survivor group than in the survivor group. However, the Cox regression model in the study by Emektar et al. showed an adjusted HR of less than 1.0 (0.997 [95% CI, 0.994–0.999]), which seems to be consistent with our findings.

Djordjevic et al. evaluated the predictive value of PLR on surgical ICU admission among 392 critically ill and injured patients. [16] In this study, critically ill patients with severe trauma were defined as having an ISS > 25. The cohort was relatively heterogeneous because the patients had different diseases, including peritonitis, pancreatitis, and trauma with or without sepsis. Hence, only a small number of patients (n = 46) formed the trauma subgroup. PLR was higher in the non-survivor group (378.19 [IQR, 237.35–574.38 vs. 220.33 [143.39–315.17], p = 0.029) than in the survivor group. There were 83 patients in the trauma with sepsis subgroup. PLR was not significantly different in the non-survivor group (246.08 [IQR, 162.22–373.41] vs. 198.60 [145.80–336.45], p = 0.356). The odds ratio or HR of PLR in the trauma subgroup or the trauma with sepsis subgroup was not documented.

This study has some limitations. First, this was a retrospective chart review study conducted in a single center. The reproducibility and generalizability of the results should be tested in a larger study. Second, there could be unknown confounding factors. We adjusted for baseline characteristics, comorbidities, physiological variables, traffic accident mechanisms, and risk scores in the regression model. However, other potential laboratory biomarkers may eliminate the significance of PLR for the outcome. [22, 23] Despite of propensity matching between survivors and non-survivors to identify the independent effect of PLR, a few variables still showed different distribution–GCS score, head/face/neck, RTS, TRISS, ICU admission, emergency operation, and platelet count. RTS, a variable considered in matching, showed a significant difference between the survivors and non-survivors, despite the absolute differences between the two groups being reduced. This point reflected that the characteristics between the two groups were much different and could not be perfectly matched. This would be inevitable considering death as a fatal outcome. The purpose of this study was to investigate the independent association between PLR and in-hospital mortality, and adjusting for variables with significant differences could resolve the differences between the two groups, despite the possibility of residual confounding factors. Third, the severity of the enrolled patients can be concern of interest. We enrolled the adult traffic accident patients, but nearly half of them (45.5%) did not use EMS to arrive the ED. And considerable difference was found regarding the EMS use (52.4% in survivor group vs. 76.7% in non-survivor group). Even though PLR performance have been assured through the propensity matching analysis in which the EMS use was controlled well, severity of the enrolled patients may be still questioned, therefore, the immune and inflammatory response may be questioned as well. Unfortunately, the present study could not exclude the patients who did not use EMS because it resulted in a large reduction of a cohort. Further large study is needed to confirm the PLR performance.

In conclusion, PLR was significantly associated with an increased risk of in-hospital mortality in admitted adult traffic accident patients. Additionally, PLR had a better prognostic value than relative thrombocytopenia or lymphocytosis. PLR can be used as a readily usable prognostic parameter with no additional cost in clinical practice. Further studies are needed to evaluate the clinical role of PLR in different experiences and in different populations.
